# Piezoelectric Size Effects in a Zinc Oxide Micropillar

**DOI:** 10.1186/s11671-015-1081-2

**Published:** 2015-10-08

**Authors:** Tao Li, Yu Tong Li, Wei Wei Qin, Ping Ping Zhang, Xiao Qiang Chen, Xue Feng Hu, Wei Zhang

**Affiliations:** State Key Laboratory of Material-Oriented Chemical Engineering and School of Chemical Engineering, Nanjing Tech University, Nanjing, Jiangsu 210009 People’s Republic of China

**Keywords:** Pulsed laser ablation, ZnO micropillars, Piezoelectric, Nanogenerator

## Abstract

In this work, the dependence of piezoelectric coefficients (PE) on the size of artificial fabricated ZnO micropillars on Si substrate is investigated. ZnO full film is grown with c-axis orientation and an average grain size of 20 nm at a substrate temperature of 500 °C by pulsed laser ablation. The micropillars with the size range of 1.5 to 7 μm are formed by top-down semiconductor device processing. The PE, characterized by piezoelectric force microscopy (PFM), is found to increase from 18.2 to 46.9 pm/V, when the ZnO pillar size is reduced from 7 to 1.5 μm. The strong PE dependence on ZnO pillar size can be explained by local changes in polarization and reduction of unit cell volume with respect to bulk values. These results have strong implications in the field of energy harvesting, as piezoelectric voltage output scales with the piezoelectric coefficient.

## Background

The fundamental principle of ZnO nanogenerator [1–3] is to utilize mechanical energy of the environment, which is available everywhere from irregular vibrations, human activity even noise with a wide spectrum of frequencies and time-dependent amplitudes. Piezoelectricity is caused by the nonsymmetrical crystal structure of certain materials, which results in an effective change in polarization in response to an applied mechanical strain. The first prototyping of a nanogenarator based on ZnO piezoelectric nanowire (NW) arrays [4–6] has been demonstrated to be able to drive microsensor and sensor network nods in micropower range. However, it is still far away from a milli-power output source, which is required by most practical applications of individual sensors and sensor network systems. The lacking of high power output in current ZnO NW generation is partially attributed to the structural properties of piezoelectric material, especially imperfect c-axis oriented crystalline of ZnO NW material and low yield in the NW device [6]; both are synthesized by chemical methods. Furthermore, in the ZnO NW, the voltage potential generated from the bent nanowire is between the left and right sides of single nanowire, which results in a fabrication and power collection dilemma because of precisely contacting two sides of the nanowire in-turn impractical [7].

The piezoelectric ZnO full film generator has emerged as a reliable high power source [8–10], considering that the voltage potential can be easily produced at the top and bottom electrode sandwiched ZnO film structure. The formation of the thin film structure is also compatible with current semiconductor processes for mass production. It provides an easy way to expand power output by integrating more units, either in parallel or series, together. An energy harvester on a single thin flexible plastic substrate enables high output performance (output voltage of 10 V and output current of 0.6 μA) for operating self-powered wireless data transmissions has been achieved [11], but still not in megawatt level [12, 13].

In principle, as the size of piezoelectric film structures is reduced to the nanoscale, the conversion efficiency can be improved dramatically due to nanomaterial’s relatively large tolerance of deformations prior to failure [14]. Recently, various experimental studies have probed either the electrical [15, 16] or mechanical [17–19] behavior of nanowires separately. Riaz et al. [20] experimentally and initially studied electrical potential-related size effects of ZnO nanowire using both the high-temperature vapor-liquid-solid (VLS) and the low-temperature aqueous chemical growth (ACG) methods. They also theoretically investigated relationship between aspect ratio and density of the nanowire and resulted electrical potential output. The challenges associated with experiments of aspect ratio vs. electrical potential are (i) difficulties in sample manipulation at the nanoscale, (ii) making appropriate electrical measurements accounting for contact resistances, and (iii) measuring currents and voltages with sufficiently high resolution. The difficulties in conducting such experiments seem to be the primary reason for large discrepancies observed in the experimentally reported piezoelectric coefficients for ZnO nanostructures.

In this work, by following Riaz’s theoretical study [20], we have experimentally investigated piezoelectric size effects of man-made ZnO micropillars. The high-oriented ZnO films are grown by pulsed laser ablation (PLD) and micropillars with different size are patterned by top-down semiconductor device processing. The piezoelectric properties of microarrays are measured by piezoelectric force microscopy (PFM). A theoretical model to explain the observed size-enhanced piezoelectric effect is also suggested.

## Methods

Targets for PLD, ranging from 93–99 % of theoretical density, are prepared from 99.99 % pure ZnO powder by cold-pressing (4 tons on a 26-mm diameter pellet) followed by sintering for 12 h at 1100 °C in air. These targets are then placed in a rotating holder and ablated using a KrF excimer (Lambda Physik COMpex 102, wavelength of 248 nm, energy of 180 mJ/pulse) laser. The fluence of 5 J/cm^2^ is kept constant for the experiments. The ablations are carried out with an oxygen background gas pressure of 0.3 mTorr for periods of 30 min at pulse repetition rates of 5 Hz. A 1 cm × 1 cm Si (100) substrate has been employed. The deposition temperature is 500 °C, and the thickness of Zn film is 800 nm. The use of 248 nm KrF excimer radiation for ZnO ablation was found in previous research to produce films of significantly higher quality than those grown using longer wavelength radiation [21–23]. Furthermore, ablation with 248-nm radiation leads to a smooth target surface after ablation [24] and, hence, all targets used here were pre-ablated prior to initial deposition, and not polished between depositions. The structure of the deposited film is performed by XRD analyses with a Philips PW3710 system (CuKα radiation, *λ* = 0.15406 nm). Surface morphology of the films is characterized by atomic force microscopy (AFM) (CSPM 5500). The optical microcopy and scanning electron microcopy are used for microsize characterization. The grain size of the deposited ZnO film is characterized by transmission electron microscopy (TEM).

**Fig. 1:**
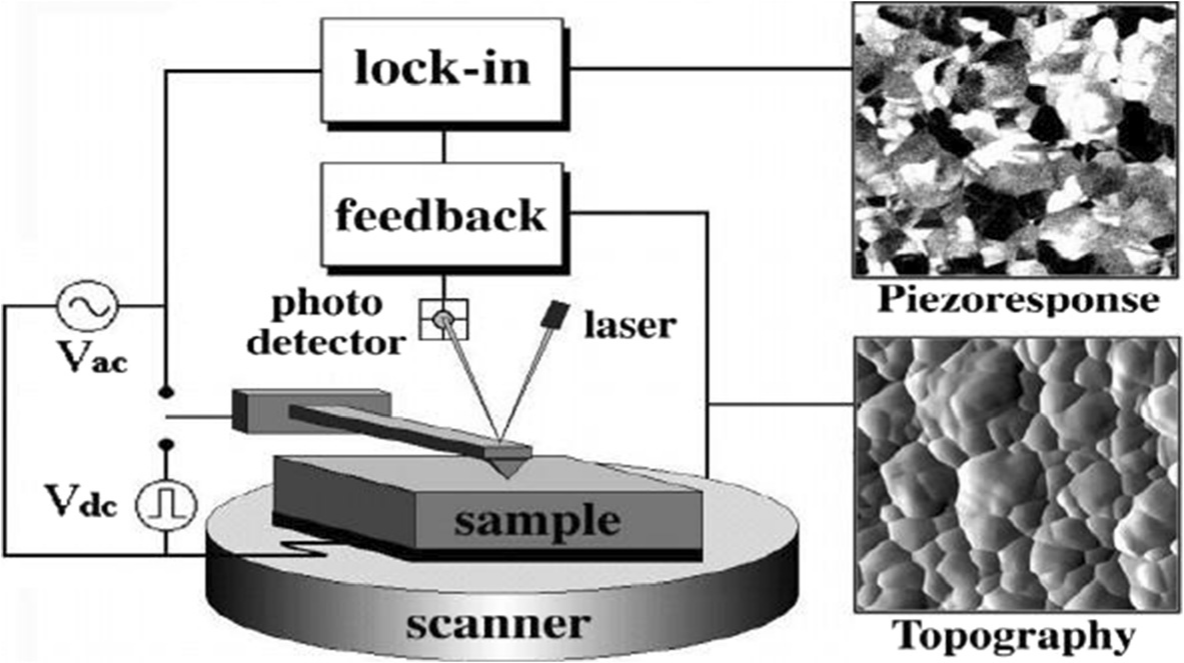
The cartoon of the PFM experimental setup

PFM is nowadays a powerful tool for investigating local electromechanical coupling phenomena on the nanoscale. PFM utilizes a basic experimental setup of AFM in which an alternating current (AC) voltage is applied between a conducting tip at the end of a cantilever and the bottom electrode of the piezoelectric sample. The AC voltage induces local oscillations of the sample surface due to the piezoelectric effect. The modulated oscillations lead to mechanical displacements of the cantilever. The recorded signals are then extracted using a lock-in amplifier technique. Details on the principle and application of this technique can be found in Fig. 1. In this study, PFM was performed in air using a commercial Oxford 4680 with a Dual AC Resonance Tracking (DART) controller that serves as both the function generator for the AC modulation bias and the lock-in amplifier. A nanoscale tip with a resonant frequency of 22 kHz and spring constant of 0.3 N/m was used. An AC voltage with a magnitude of 1.5 V and a frequency of 20 kHz was applied between the conductive Pt -coated Si tip and the Mo bottom electrode of the sample.

ZnO micropillars with the size range of 1.5 to 7 μm are fabricated by using a typical top-down semiconductor device processing technique. ZnO/Mo multiple layers are initially deposited by sputter and PLD, respectively. A 50-nm Mo metal layer is mainly served as a bottom contacting electrode for PFM characterization. After the photoresist spin and bake, UV exposure, and chemical developing, a 1.5 to 7 μm pillar pattern is printed on the surface of the ZnO/Mo. Then, an ion mill process is utilized to transfer the resist pattern into the ZnO and stop at Mo layer. After the resist stripe, micropillars of ZnO are formed.

## Results and Discussion

**Fig. 2:**
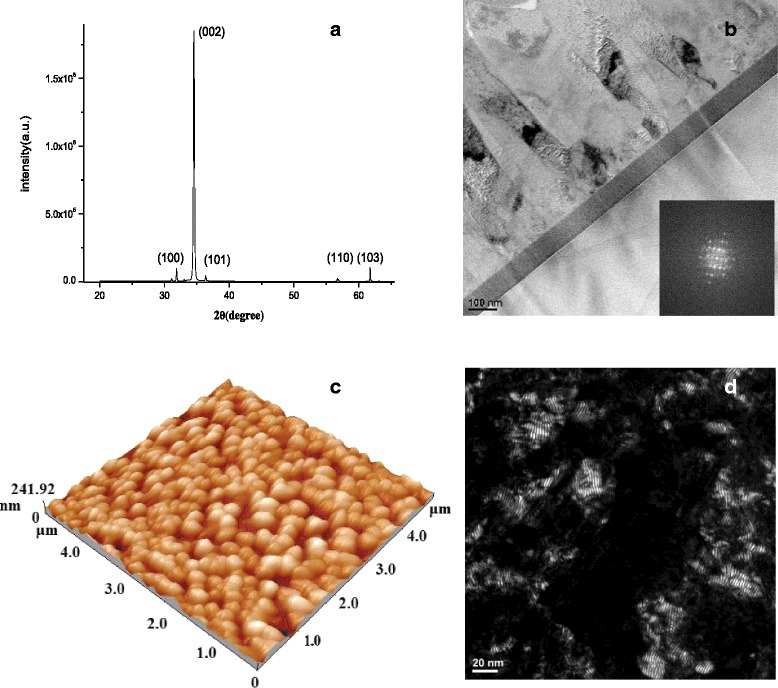
**a** XRD patterns of ZnO films grown on Si(100) substrates at 500 °C, **b** cross-sectional dark-field TEM images of the ZnO film, **c** AFM images, and **d** dark-field TEM

**Fig. 3:**
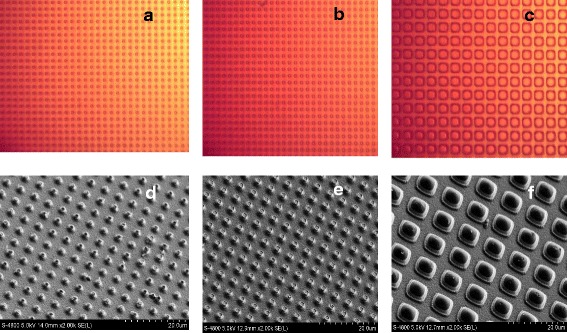
Optical microscope images of ZnO micropillars: **a** 1.5 μm, **b** 3 μm, **c** 7 μm, and 45° and tilted SEM images of ZnO micropillars: **d** 1.5 μm, **e** 3 μm, and **f** 7 μm

XRD was used to characterize the crystal structure of the ZnO thin films deposited on Si (100) substrates at growth temperatures of 500 °C. Figure 2a shows a typical XRD pattern for the film. One main peak occurs at 34.5°, and three small peaks appear at 31.8°, 36.2°, and 56.6°. The former is attributed to the diffraction from the ZnO (002) plane, while the latter three correspond to the diffractions from the ZnO (100), (101), and (110) planes, respectively [25]. The XRD spectra of the ZnO samples exhibits a strong (002) peak, which indicates that they have a preferential growth orientation along the c-axis perpendicular to the substrate surface. Using the Scherrer formula, *D*_XRD_ = 0.94*λ*/(*β*cos *θ*_β_), where *λ*, *θ*_β_, and *β* are the X-ray wavelength, Bragg diffraction angle, and the line width at half maximum of the diffraction peak, respectively, the mean crystallite sizes (*D*_XRD_) can be calculated to be 41.2 nm for the films grown at 500 °C. Figure 3b shows the cross-sectional dark-field TEM micrographs of the ZnO film on a Si/Mo substrate deposited at 500 °C. The thickness of the film obtained from TEM is approximately 800 nm. Columnar growth can be clearly observed for the film deposited on the Si/Mo substrate. The shape of the grains in the upper region of the structure is convex. This indicates that the upper region is more stable and closer to equilibrium morphology due to the atomic mobility and stress relaxation in the upper region being almost fully achieved compared to in the lower region [26]. The inset in Fig. 2 shows a selected area electron diffraction where the electron beam is parallel to the [21–30] zone axis of the ZnO. The indexed diffraction pattern confirms the hexagonal structure of the ZnO thin film. The dark-field cross-sectional TEM micrograph shows that the growth pattern of the film is columnar, with alternate nonuniform bright and dark columns.

Figure 2c shows the AFM images of the ZnO thin film deposited at a substrate temperature of 500 °C. The films are uniform, dense, and well packed between particles. The films shows the columnar structure, and the particles are arranged uniformly. The average particle size (DAFM) of the films was approximately 39 nm, as calculated from AFM images. During the deposition, there is a possibility that the oxygen atoms bond together and are pumped out as O_2_ gas. It has also been reported that the oxygen gets re-evaporated from the surface at higher substrate temperatures [27]. The root mean square surface roughness (*Rq*) was calculated to be 3.14 nm for the films at a 500 °C substrate temperature. The TEM analysis provides measurements of the average grain size with a data accuracy of ±0.1 nm. Figure 2d shows the bright-field TEM micrographs of the ZnO thin films with an average grain size of 42 nm. The diffracting grains exhibiting the darkest contrast indicate the typical grain sizes and shapes. Because these images show only grains with a particular crystallographic orientation, it is easier to identify individual grains. While the grain sizes observed in the dark-field images of all samples typically were in close agreement with the calculated d_XRD_ values, a few grains in the dark-field image (e.g., Fig. 2d) appeared to be much larger, possibly due to the overlap of the bright contrast arising from multiple diffracting grains of similar crystallographic orientations [28]. Figure 3a–f presents the images of a ZnO micropillars fabricated by top-down device processing and characterized by an optical microscope and SEM. Microarrays exhibit a uniform pillar distribution at all sizes ranging from 1.5 to 7 μm.

**Fig. 4:**
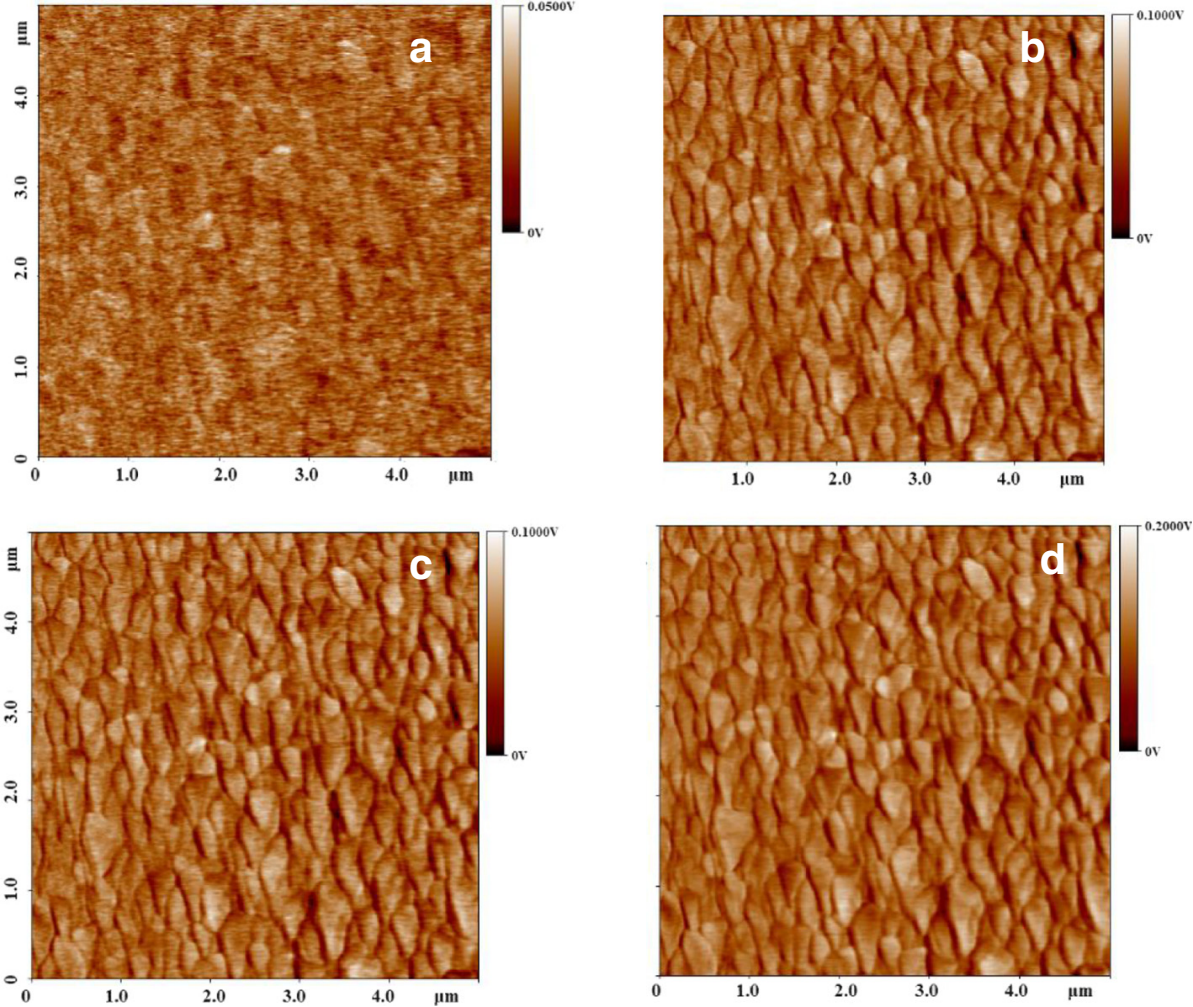
PFM phase image for a ZnO pillar with 7 μm in size under different applied voltages, **a** 0.5 V, **b** 1 V, **c** 1.5 V, and **d** 2 V

The effect of ZnO size on piezoelectric properties at a range of 1.5–7 μm pillars is characterized by PFM under standard piezoelectric phenomena [29, 30]. In the piezoelectric measurements, the interaction between the tip and electric field was ignored [31]. Figure 4a-d shows morphologic profile of a ZnO pillar of 7 μm under different polarized voltages from 05 to 2 V. The PFM phase image exhibits sharp dark and light contrast. Dark and light domains correspond to grains with a downward and upward spontaneous polarization orientation, respectively. Note that most of the grains are light colored. This means that the spontaneous polarization orientation of most of the grains is upward.

**Fig. 5:**
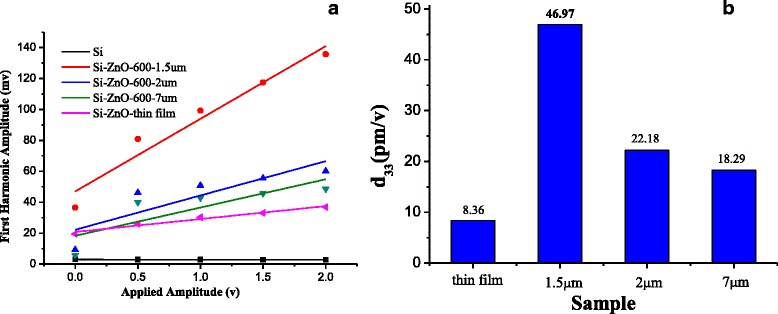
Characterization of the ZnO film and micropillars in the size range of 1.5 and 7 μm piezoresponse: **a** linear fitting of the displacement vs. applied ac voltage at various pillar sizes and **b** piezoelectric coefficients, d_33_, calculated from the slopes for the ZnO micropillars in the size range of 1.5 to 7 μm

To reference the piezoelectric response to the piezoelectric material, the piezoelectric coefficients (PE) response on a bare single crystalline Si (100) substrate was also characterized. So, the impact of the initial film roughness from the substrate on the PE result can be subtracted. The displacement as a function of applied voltage for the films is shown in Fig. 5a. The piezoelectric coefficient, *d*_33_, can be deducted from the slope of the resulting amplitude of the displacement vs*.* applied voltage plot, which refer to both peak-to-peak values of displacement and applied voltage, respectively. The piezoelectric efficiency, *d*_33_, was calculated using the follow equation [32].

1 $$ {d}_{33}={A}_0/{U}_0 $$

where *A*_0_ is the vibration amplitude and *U*_0_ is the amplitude of the testing ac voltage. As summarized in Fig. 5b, the piezoelectric constant, d_33_, is approximately 0.26 pm/V on a bare Si substrate, which is similar (*<*0.5 pm/V) to literature values [23]. The PE of full ZnO film is about 8.6 pm/V film. In contrast, piezoelectric constants at the ZnO micropillars continuously increases from 18.2 pm/V to 46.9 pm/V when the pillar size is reduced from 7 to 1.5 μm.

To further understand the origin of this PE size dependence, the effect of atomic restructuring is considered to decouple from the absolute value change of polarization. Ravi et al. [14] calculated the polarization (per atom) as a function of strain for GaN nanowires of different diameters using PSP1 and the PBE functional. It is noteworthy that the absolute value of polarization for nanowires is smaller than that of bulk. On the contrary, when the polarization per unit volume is plotted, the trend is reversed. This asserts that the reduction in volume of nanowires due to restructuring of the surface atoms (surface reconstruction) plays a significant role in enhancing the piezoelectric properties of the nanowires. The reduced dipole moment with respect to bulk, as observed for nanowires, is in general agreement with the reduced polarization for nanowires [33]. However, the volume of nanowires is smaller compared to the bulk value. This plays an important role in enhancing the piezoelectric coefficient, which depends on the polarization per unit volume.

On the other hand, Riaz et al. [20] studied the electrical potential vs. the aspect ratio of different nanowires at a fixed length of 1000 nm and varied diameters. It is found the output electrical potential increases with decreasing in diameter of the nanowires and saturated at a diameter below 12.5 nm. It is explained that as the aspect ratio increases, the deflection of the nanowire increases, leading to an enhancement in the output electrical potentials. Upon further increase in the length (increase of the aspect ratio), the output voltage signal starts to decrease due to the excessive deflection in the nanowire in both the lateral and the vertical directions, which may cause a screening of the charge carriers on the outer surfaces of the nanowires resulting in a decrease of the electrical potential. Our experimental results, i.e., PE increases with a decreasing in the diameter of ZnO pillar, are consistent with Ravi’s theoretically observations.

The charge redistribution and interatomic rearrangement in ZnO nanowires is another important factor in affecting polarization [34, 35]. The charge deviates from bulk behavior is primarily on the surface of the nanowires. The overall charge redistribution and interatomic rearrangement in the axial direction of nanowires have the net effect to reduce polarization. However, the contraction in the radial direction, due to surface relaxation, leads to a reduction in overall nanowire volume with respect to a bulk crystal with the same number of atoms. This reduction in volume, in essence, causes the observed enhancement in piezoelectric coefficients.

In our experiment, ZnO pillar size decreased from 7 to 1.5 μm. Based on the charge distribution analysis and calculation of first-order dipole moments, overall polarization is found to be reduced in nanowires. However, the piezoelectric coefficients are found to be much higher due to surface relaxation which induced volume reductions in micropillar. The study highlights a direction of how to enhance the piezoelectric effect and increase power output.

## Conclusions

The size dependence of piezoelectric coefficients in a ZnO micropillars was investigated. ZnO full film was grown with c-axis orientation by pulsed laser ablation. The piezoelectric constant, characterized by PFM, increased from 18.29 to 46.97 pm/V, when the ZnO pillar size at the microarray reduced from 7 to 1.5 μm. The observed size effect can be explained by local changes in polarization and reduction of unit cell volume with respect to bulk values. The findings reported here, therefore, suggest new way to enhance piezoelectric power output for self-powered sensor network systems.
